# Accelerating On-Device Learning with Layer-Wise Processor Selection Method on Unified Memory

**DOI:** 10.3390/s21072364

**Published:** 2021-03-29

**Authors:** Donghee Ha, Mooseop Kim, KyeongDeok Moon, Chi Yoon Jeong

**Affiliations:** Human Enhancement & Assistive Technology Research Section, Artificial Intelligence Research Laboratory, Electronics Telecommunications Research Institute (ETRI), Daejeon 34129, Korea; dhha@etri.re.kr (D.H.); gomskim@etri.re.kr (M.K.); kdmoon@etri.re.kr (K.M.)

**Keywords:** deep learning acceleration, processor selection algorithm, on-device learning, acoustic scene classification, mobile devices

## Abstract

Recent studies have applied the superior performance of deep learning to mobile devices, and these studies have enabled the running of the deep learning model on a mobile device with limited computing power. However, there is performance degradation of the deep learning model when it is deployed in mobile devices, due to the different sensors of each device. To solve this issue, it is necessary to train a network model specific to each mobile device. Therefore, herein, we propose an acceleration method for on-device learning to mitigate the device heterogeneity. The proposed method efficiently utilizes unified memory for reducing the latency of data transfer during network model training. In addition, we propose the layer-wise processor selection method to consider the latency generated by the difference in the processor performing the forward propagation step and the backpropagation step in the same layer. The experiments were performed on an ODROID-XU4 with the ResNet-18 model, and the experimental results indicate that the proposed method reduces the latency by at most 28.4% compared to the central processing unit (CPU) and at most 21.8% compared to the graphics processing unit (GPU). Through experiments using various batch sizes to measure the average power consumption, we confirmed that device heterogeneity is alleviated by performing on-device learning using the proposed method.

## 1. Introduction

Recent developments in computing hardware (e.g., graphics processing units (GPUs) and tensor processing units (TPUs) [1]) have enabled large scale parallel processing, resulting in a substantial reduction in the inference/training time for deep learning on PC/server platforms. As hardware performance improvements have made neural network models deeper and wider, the deep learning model has outperformed humans in various fields such as computer vision, natural language processing, and audio classification [2,3,4,5,6]. Many recent studies have used the superior performance of deep learning algorithms, which normally run on PC/server platforms, for their deployment in mobile devices [7,8,9,10,11]. However, there are several problems to be solved before deep learning applications can be run on mobile devices.

Device heterogeneity is one of the main challenges encountered when using a mobile device in the training network model, and it is caused by the differences between mobile device sensors used to collect the data. The differences between sensors used to gather training data and those used to classify the test data can cause performance degradation in the classification model. For example, in the field of acoustic scene classification (ASC), the classification performance differs by more than 10% depending on the influence of the microphone used to collect sound [12,13,14,15]. To solve this issue, it is desirable to train a network model using the data collected by the sensor of the mobile device.

Training the network model on mobile devices can generally be divided into centralized deep learning and on-device learning. Centralized deep learning is a method of transmitting data from a mobile device to a server, training it using the server, and transmitting the model trained using the server to the mobile device. In contrast, on-device learning performs retraining using the data on the mobile device itself. In the case of centralized deep learning, there are various issues to address such as privacy and communication [16,17]. To overcome these issues, there is an increasing demand for a shift in training networks from centralized learning to distributed on-device learning.

Another related issue to consider is the limited computing power and memory traditionally associated with mobile devices. Compared to a desktop computer, mobile devices have insufficient resources to execute the computationally intensive tasks required for deep learning training. Therefore, existing studies applying deep learning algorithms to mobile devices mainly focus on accelerating the deep learning inference, which requires relatively low computing power. To accelerate the deep learning inference, existing approaches optimize inference for mobile processors [18,19,20] or perform inference by dividing the model across multiple computing resources of the mobile device [18,21,22,23,24,25]. Other approaches focus on enhancing the usability of memory to eliminate data copy time [22,23]. However, the existing approaches for accelerating deep learning inference cannot directly be applied for accelerating deep learning training because training the network model involves more complex components.

**Figure 1:**
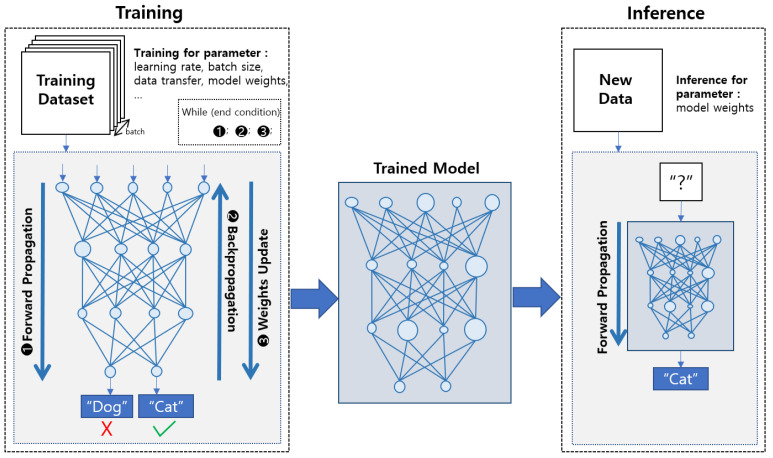
Conceptual illustration of the process of training and inference.

Deep learning training is more complex than inference because each layer has many functions and requires data transfer, as depicted in Figure 1. Deep learning inference is acyclic because it only performs forward propagation (FP) with the trained model. In contrast, training is a cyclical process, which includes FP, backpropagation (BP), and weights update (UP), whereby the model is trained. The existing method, which efficiently utilizes the computing resources and memory of a mobile device for deep learning inference/training, does not simultaneously consider data transfer and the cyclic process of on-device learning. Therefore, a new approach to accelerate deep learning training on mobile devices is necessary.

To address these challenges, herein, we propose a layer-wise processor selection method (PSM) on unified memory, accelerating deep learning training by efficiently using the computing resources (i.e., CPU and GPU) and memory of mobile devices. Our work presents a method for the efficient use of unified memory and a method of selecting a processor for each layer that can maximize training efficiency. The unified memory of mobile devices can be accessed by the CPU and GPU simultaneously, minimizing the overhead of transferring data between processors during the training process. The proposed method leverages these systemic features of mobile devices. Although the overhead of data transfer caused by mapping/unmapping on unified memory can be ignored in the inference step, the overhead of mapping memory to access data occurs when the processors of FP and BP are different during neural network training. Therefore, the training step of the deep learning model has a layer-wise dependency on data transfer. The proposed layer-wise PSM selects a processor by comparing the mapping/unmapping overhead and latency per layer including FP, BP, and UP. Therefore, layer-wise PSM can consider the characteristics of unified memory and the cyclic process of deep learning training and can lead to the acceleration on the network’s model training.

Specifically, this study makes the following contributions:

- We propose the layer-wise PSM on unified memory to effectively utilize memory and resources. Compared to existing methods for inference, the proposed method is made more suitable for deep learning training by considering its cyclic process;
- We explore the usability by applying on-device learning to the ASC field. The performance of on-device learning can be varied by various factors such as batch size and average power consumption. Through experiments using various batch sizes to measure the average power consumption, we confirm that device heterogeneity, which is a challenging issue of ASC, is alleviated by performing on-device learning using the proposed method.

The remainder of this paper is organized as follows. Section 2 reviews the related works. Section 3 presents a detailed description of the proposed layer-wise PSM on unified memory. The experiments and results are presented in Section 4. Finally, the conclusions and discussions are presented in Section 5.

## 2. Related Work

### 2.1. Accelerating Inference/Training of Neural Network on Mobile Devices

Many studies have been conducted on the acceleration of deep learning inference on mobile devices consisting of heterogeneous processors. One approach to accelerate the deep learning inference is to optimize the deep neural network for use with a heterogeneous processor. DeepMon [20] is a system to optimize and execute large deep neural networks on mobile devices at a low latency. Cappucino [18] has proposed efficient parallel processing technology and the optimization of the convolutional layers of deep neural networks for a mobile SoC (system on a chip). In another study, to accelerate the inference, CNNdroid [19] and Deepear [26] optimized the application of deep learning using mobile GPU and a digital signal processor (DSP), respectively.

Considering the effective use of the available computing power of mobile devices, some reports propose methods to execute each layer of the network on different processors. DeepX [21] and Mosaic [25] distribute the execution of model layers to different computing resources such as the CPU, GPU, DSP, or neural processing unit (NPU). In contrast, μlayer [22] proposed the execution of a single neural network layer using both the CPU and GPU. All the approaches used in previous works [18,19,20,21,22,25,26] are limited in that they only focus on accelerating the inference by optimizing the neural network model or efficiently using multiple processors of mobile devices.

From a fundamental point of view, methods making efficient use of the shared memory of mobile devices have been proposed [22,23]. These studies used shared memory to eliminate the data copy time between the CPU and GPU [22] or prevent data duplication for the GPU [23]. However, it can only prevent the duplication of memory or eliminate the data copy time.

The implementation of deep learning training is more complex than that of deep learning inference owing to a lack of resources and the complexity of the process. To solve this issue, a study on deep learning training on mobile devices (DeepMobile) has been conducted [23,24,27]. DeepMobile [23,27] utilized shared memory to solve the memory shortage during training and to optimize mobile GPUs to accelerate training on mobile devices. Another study profiled the latency, data copy time, and search processor pathing using dynamic programming without shared memory [24]. However, these previous studies either only use the GPU to execute training or do not use shared memory, resulting in poor memory efficiency.

### 2.2. Hardware for Accelerating Neural Network

To improve the speed of deep learning inference/training, several studies have used dedicated hardware accelerators [1,28,29,30]. An architecture that uses memory efficiently for automatic and flexible optimization has been proposed [30]. Studies on DianNao [31] have proposed various neural network accelerators. Google implemented the TPU, which is the hardware optimized for deep learning operations, by applying the systolic array to the hardware [1]. However, previous studies are limited to specific purposes and specific hardware. Thus, in addition to existing methods requiring cost and effort to manufacture special hardware, because the hardware is designed for a specific purpose, it also has inferior flexibility.

### 2.3. Acoustic Scene Classification (ASC)

To show that on-device learning can solve a device heterogeneity, we used ASC, because it allows the performance of the model to vary depending on the device that recorded the sound. ASC is the task of identifying a scene as one of a set of pre-defined classes from a recorded audio signal. It has gained considerable interest in recent years owing to its diverse applications. The traditional classification method for ASC uses the support vector machine (SVM) and Gaussian mixture model (GMM). This method provides an acceptable classification performance but does not have the high-level feature abstraction capability of deep learning methodologies. Recently, some neural networks with deep architecture applied to ASC have been proposed [32,33].

A recent trend in the field of ASC is to adopt data driven methods, wherein acoustic scene features are learned from data [34]. Among the convolutional neural network (CNN) models, the ResNet model [35] exhibits high accuracy and thus is typically used as a backbone neural network model [36,37,38,39]. A crucial event in this field is the DCASE (detection and classification of acoustic scenes and event) challenge, which deals with the various tasks to tackle the issues of the ASC. Among the various tasks of DCASE, task 1.A deals with the issue of device heterogeneity. The dataset of task 1.A consists of audio scene samples recorded from multiple devices as well as simulated sound. The test data are used to evaluate the generalized performance on sound recorded from unknown devices. The results of task 1.A indicate that it is difficult to classify sounds recorded with different devices even if they are in the same scene.

## 3. Proposed Method

The neural network model consists of multiple layers, and each layer can have multiple steps, such as FP, BP, and UP, during network model training. The inference stage of the network model has the same number of layers and steps, whereas the training stage of the network model may have 2 or 3 times as many steps per layer and requires more computing power than the inference stage. Therefore, to train a network model on mobile devices that have limited resources, it is necessary to develop a method that can use memory and computing resources efficiently.

The existing methods [21,22] focus on minimizing the processing time of each step and do not consider the data transfer time. When the processors used for the FP and BP steps are different in the same layer, the total latency of training is affected by memory access time caused by the data copy. The existing methods do not consider this overhead and cannot optimize the total latency of network model training. Therefore, we propose a layer-wise PSM on a unified memory method to optimize the total latency of network model training considering the overhead due to the processor difference in the same layer. In this section, we describe the data transfer on unified memory and layer-wise PSM.

### 3.1. Data Transfer on Unified Memory

**Figure 2:**
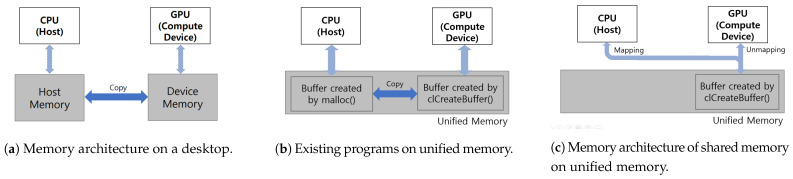
Memory architecture comparison of desktop platforms and mobile devices.

Compared to a desktop computer, mobile devices have limited resources and memory. Deep learning inference typically takes a single piece of data as an input and requires a small amount of memory for the model. Typically, neural network model training processes *N* data at once to use memory efficiently; thus, training requires more memory than inference. When using *N* training data simultaneously, deep learning training requires *N* times more memory and data than inference. NVIDIA GPUs, which are used for deep learning training on desktop computers, have access to both host memory and device memory. As depicted in Figure 2a, because the desktop computer has a separate memory for the CPU and GPU, performing an operation on the GPU inevitably requires a data copy, which leads to high latency due to the limited bandwidth of data transfer.

In contrast, mobile devices have a unified memory without physical memory separation, as depicted in Figure 2b,c. When performing operations on a different processor, the existing programs and framework, such as Tensorflow, Darknet-OpenCL, and pyTorch, use copying to transfer the data, which is an inefficient process. For example, to perform on the GPU after finishing execution on the CPU, the data used by the CPU must be transferred to the GPU via copying. Figure 2b shows a data copy between the CPU and GPU on unified memory. However, the proposed method using unified memory transfer data for use by one processor is also to be used by a different processor by transferring access to allocated memory addresses, as depicted in Figure 2c. Our method simply changes the accessibility of physical memory addresses. Thus, the proposed method can increase the efficiency of memory usage by preventing memory duplication and data copy. Therefore, the proposed method can train the network model with a larger number of layers and a larger batch size and can reduce the latency of training by eliminating the data-transfer overhead. In addition, the proposed method devises a selection method to find an optimal processor for each layer for model training on a mobile device using unified memory.

### 3.2. Layer-Wise Processor Selection Method (PSM)

**Figure 3:**
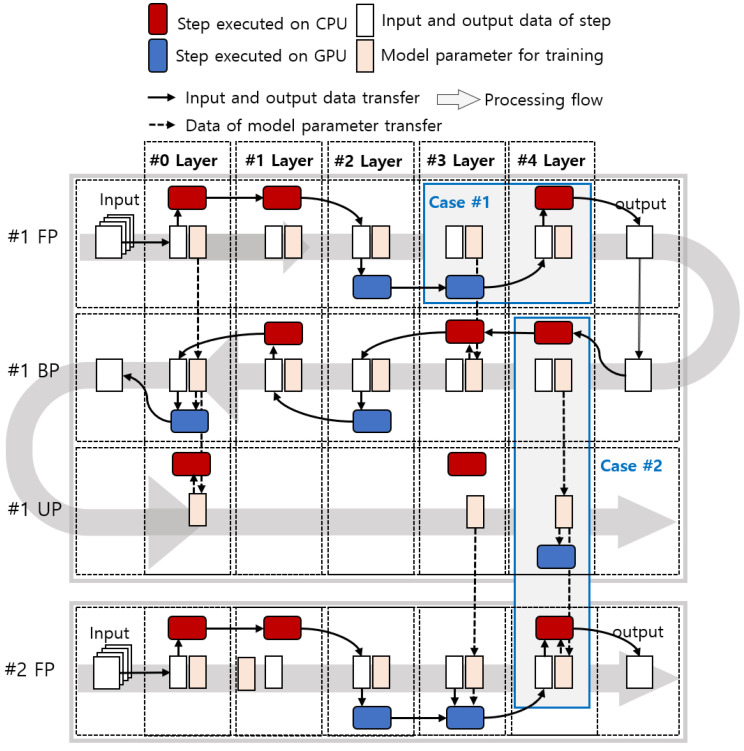
Illustration of the on-device learning with data transfer.

The latency of each step of the neural network varies depending on the characteristics of the layer, size of the filter, and size of the input data. When performing deep learning inferences, ResNet-18 performs the same number of steps as the number of layers. ResNet-18 [35], which has 31 layers by separating maxpool and shortcut into different layers, is performed in 31 steps. In contrast, deep learning training requires 82 steps (32 FP, 32 BP, and 18 UP) because convolutional layers and fully connected layers have a weight update step. The existing methods accelerate the deep learning inference by selecting a processor with low latency for each layer, and these methods can be applied to accelerate the deep learning training. When performing deep learning inference/training on a mobile device, data must be copied between processors. In addition, as the number of steps increases in the process of training a network model, data transfer occurs more frequently, and the overhead increases. Therefore, we need to consider the overhead of data transfer for model parameters and the output of executing steps, as depicted in Figure 3.

Because training is performed step by step, adjacent steps and layers are affected by the data transfer of model parameters and training data. Overhead due to data transfer occurs in two cases: 1. Processors of the current step and the next step are different. Step-wise input and output data transfer is required. 2. Processors performing FP, BP, and UP in the same layer are different.

In case 1, the output of the current step is the input data of the next step, as depicted in case 1 of Figure 3. In case 1 of Figure 3, the step processors of the fourth and fifth layers are the GPU and CPU, respectively. Since the step processors of the fourth and fifth layers are different, the output data of the fourth layer step must be copied from the GPU memory to CPU memory.

Case 2 occurs when FP, BP, and UP are executed by different processors based on the layer, as depicted in case 2 of Figure 3. In case 2 of Figure 3, the processors of #1 BP of the fifth layer and #1 UP of the fifth layer are the CPU and GPU, respectively. To update weights and execute training, data of the model parameters must be copied from the CPU memory to GPU memory. Additionally, in #1 UP and #2 FP of the fifth layer, the data of model parameters must be copied from the GPU memory to CPU memory for training.

In deep learning inference, case 2 is not considered because the model weight and bias do not change. Unlike the inference process, parameters such as model weight and bias cyclically affect the next training operation in the same layer. The result of FP is used in BP, and the result of BP is used in UP. The updated weights and bias are used in FP, so that it can be trained with the next dataset, as depicted in case 2 of Figure 3. Therefore, it is necessary to cyclically consider the data copy for each layer.

The method with unified memory does not copy data but instead uses the mapping and unmapping function to change the memory access. In deep learning inference, case 1 is ignored because the variables that need to be executed as input and output are small [22,23,27], and case 2 does not occur because there is no change in the model. However, in deep learning training, though case 1 can be ignored, case 2 has many variables, resulting in mapping and unmapping overhead, which should be considered. Therefore, we focus on case 2, which can significantly impact the latency of network training. Each of FP, BP, and UP can be performed by either the CPU or the GPU, but they are always performed in the following order: FP, BP, and UP. Therefore, there are 8 possible permutations of the processors used in that order. In this study, the cases are identified with three letters according to the order of FP, BP, and UP, with C meaning CPU and G meaning GPU. Therefore, the 8 cases include CCC, GGG, GGC, GCG, GCC, CCG, CGC, and CGG. For example, CGG means that the processor for thee FP is the CPU, the processor for the BP is the GPU, and the processor for the UP is the GPU. To select a processor for each layer for the proposed layer-wise PSM, we profile 8 layer-wise latencies, data copy time, and overhead of mapping/unmapping.

Algorithm 1 presents the pseudo code for layer-wise PSM that determines the combinations of processors by layer. In the loop (Lines 4–19), layer-wise PSM iterates the layer from the first layer to final layer. The shortest time for each layer is compared using the result of profiling in 8 cases of data copy. It is divided into cases with and without a weight update. Line 7 is for the weights update, and the fastest combination of processors per layer is selected, compared to the latency and the 8 data copies profiled per layer. Line 14 is for the case without the weight update. The proposed method compares latency and four cases (CC, GG, CG, and GC) and selects the processors. After iteration from layer 0 to the final layer, layer-wise PSM returns the optimal combination of processors by layer.
**Algorithm 1** Layer-wise PSM1:l.latency: Execution time on CPU or GPU per layer2:l.FPBPdatacopy: Data transfer time on FP and BP per layer3:l.UPdatacopy: Data transfer time on UP per layer4:**for** i=0→net.numberOfLayers **do**5:    l=net.layers[i]6:    min=∞7:    **if** l.updateisTrue **then**8:        l.processor=09:        **for** j=0→8 **do**10:           temp=l.latency[i]+l.FPBPdatacopy[j]+l.UPdatacopy[j]11:           **if** min>temp **then**12:               min=temp13:               net.processors[i]=j14:    **else**15:        **for** j=0→4 **do**16:           temp=l.latency[i]+l.FPBPdatacopy[j]17:           **if** min>temp **then**18:               min=temp19:               net.processors[i]=j **return** net.processors

The existing methods with shared memory only considers the latency of the processor, whereas layer-wise PSM considers the data transfer of model parameters in the same layer in deep learning training. Layer-wise PSM with shared memory is effective in both deep learning training and inference considering latency and overhead of mapping/unmapping. Additionally, the layer-wise PSM can effectively reduce the training time of the deep learning model because it selects the processor with the lowest latency for each layer when using the mapping/unmapping on shared memory.

## 4. Experiments

### 4.1. Experimental Setup

**Figure 4:**
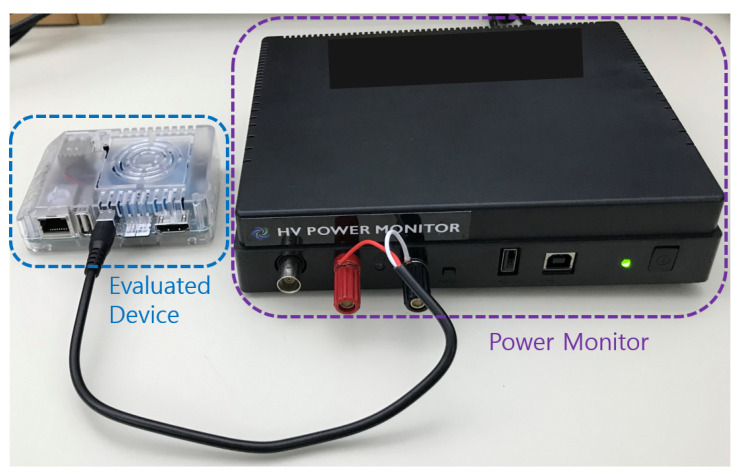
Evaluated ODROID-XU4 and high voltage power monitor (HVPM) to measure energy consumption.

To evaluate the effectiveness of layer-wise PSM on unified memory, we measured latency, data copy time, overhead of mapping/unmapping, and average power on an ODROID-XU4 computing device [40]. As a target platform for our experiment, we used the ODROID-XU4 because it has unified memory, has performance that is similar to a smartphone, and is suitable for heterogeneous computing due to the performance balance between the CPU and GPU. The ODROID-XU4 is equipped with a Samsung Exynos 5422 [41] that consists of a four big cores, ARM Cortex-A15 up to 2 Ghz, and four small cores, ARM Cortex-A7 Octa core CPUs upto 1.4 GHz, and Mali-T628 MP6 GPU. The Mali-T628 MP6 GPU offer key API support for OpenCL 1.2 Full Profile. The Exynos 5422 has 2 Gbyte LPDDR3 RAM, which is a unified memory that both the CPU and GPU can physically access in the same location, and support Linux Kernel 4.14 LTS. To measure the average power of the mobile device, we utilized the high voltage power monitor (HVPM) [42], as depicted in Figure 4.

To implement our proposed method, we modified the Darknet-OpenCL framework [43], which is an open-source framework ported from Darknet [44] using CUDA [45] to OpenCL [46]. To accelerate matrix multiplication, which takes up most of the time in deep learning operations, we modified the basic linear algebra subprograms (BLAS) library of Darknet-OpenCL, OpenBLAS [47] for CPU, and CLBlast [48] for GPU. OpenBLAS is a BLAS library optimized for specific processor types and supports acceleration through multiple threads. CLBlast is a BLAS library based on OpenCL optimized for various OpenCL devices from different vendors. We modified Darknet-OpenCL to allow both the CPU and GPU to be used, whereas the unmodified Darknet-OpenCL only allows the use of a single processor. Our modification eliminates data redundancy in the unified memory and allows the unified memory to be used by the CPU and GPU simultaneously. We implemented a function to profile the CPU and GPU execution time and the transfer time between processors. After profiling is finished, the model is trained by finding the layer-wise optimal processors.

**Figure 5:**
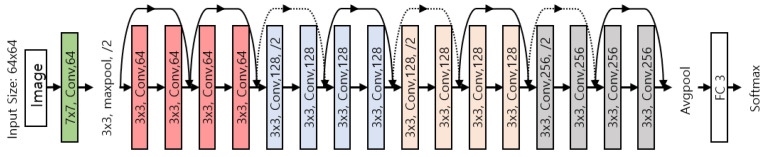
CNN (convolutional neural network) architecture of modified ResNet-18. We applied weighted sum pruning.

We used a ResNet model, which typically performs well in ASC. To use the model on mobile devices, which typically have insufficient resources, the small model used ResNet-18 and applied weighted sum pruning. The architecture structure we used is shown in Figure 5. In detail, the proposed method performs efficiently by dividing the network model operation, where the 18 layers of ResNet-18 are divided into 31 layers by dividing maxpool, avgpool, and shortcut softmax into one layer.

### 4.2. Experiments of Device Heterogeneity in ASC

To investigate the mitigation of device heterogeneity by applying the proposed method to the ASC task, we used the dataset of DCASE Task 1.A [49]. The dataset is recorded from 14 cities and by various devices (3 real devices, 6 simulated devices). To show device heterogeneity, we selected the data recorded by device A and device B. The device A dataset consists of 10,215 training data and 330 verification data. The device B dataset consists of 750 training data and 330 verification data. The dataset comprises audio with a 44.1 kHz sampling rate. To compress the input size, we took 13,850 FFT points with 50% overlap, and calculated the log-Mel spectogram. The result of a log-Mel spectogram with 64 frequency bins is 64 frames. We calculated deltas and delta-deltas from the log-mel spectogram. The result of the input size calculated from the audio signal is 64 × 64 × 3. We labeled both the device A dataset and device B dataset as 3 classes of indoor, outdoor, and transportation from 10 classes.

To perform deep learning training on a mobile device, we used ResNet-18, the effectiveness of which has already been verified in the ASC field [36,37,38,39]. We reduced the size of the ResNet-18 model, cutting the output of the convolution layer in half by applying the weighted sum pruning method [50]. The pruned ResNet-18 models used a stochastic gradient decent (SGD) optimizer with a constant learning rate of 0.001 and batch size set to 64. The number of epochs was set to 300. Our experiments were conducted using the Darknet-OpenCL [43] framework.

To solve the issue of device heterogeneity, we used transfer learning [51]. After we trained the model on the dataset from device A on the server, we deployed the model to a mobile device. We used the dataset from device B to retrain the model. In the model with the device A dataset, we used 10,215 training data and 330 validation data. For the retraining model, the device B dataset consisted of 750 training data and 330 validation data.

The model trained on the device A dataset achieved 70.12% accuracy and was created by training at the server with the data recorded by device A. When the model trained on the device A dataset was verified with the device B dataset, the accuracy was 50.12%. Additionally, the model trained on the device A dataset was retrained on the device B dataset. As a result, an accuracy of approximately 60.22% was confirmed. The accuracy of model verification on the device B dataset was 20% lower than that on the device A dataset. By verifying the model on datasets recorded by different devices, we confirmed device heterogeneity. After retraining the model on datasets recorded by different devices, we confirmed that on-device learning can solve this issue.

### 4.3. Experiments of Proposed Method

**Figure 6:**
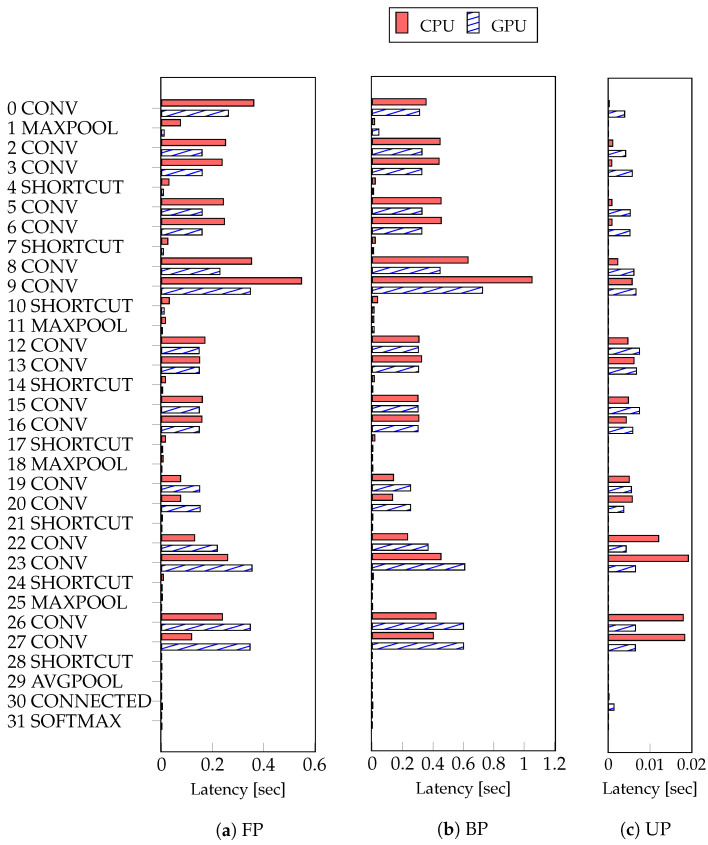
The result of profiling the latency performed by the CPU and GPU of each step. Forward propagation (FP); backpropagation (BP); and weights update (UP).

To investigate the deep learning training workload, we profiled the latency of each step in the network model training. In FP, BP, and UP, we confirmed that the latency of each step is different depending on the characteristics of the layer, the size of the filter, and the size of the input data [22,24,25]. We profiled the execution time of the CPU and GPU while training the ResNet-18 model, as depicted in Figure 6. With the result profiled, we could determine which processor had lower latency. Each layer shows different execution times on the CPU and GPU. Sincce mobile GPUs have a similar performance to CPUs, processor execution time varies depending on the characteristics of each layer. In the convolutional layers between 0 and 16, the latency of the GPU is lower than the latency of the CPU, as depicted in Figure 6a. In the maxpool and shortcut layers, the latency of the GPU was lower than the latency of the CPU. Additionally, the latency of the CPU in the avgpool and softmax layers was lower than the latency of the GPU. With the result of profiling, the existing method selects the processor with lower execution time for each layer. As depicted in Figure 6b,c, we can determine which processor has a lower execution time in the same way as the execution time was checked for BP and UP. Since the training time is mostly occupied by FP and BP, and the time of UP is less than 0.02, the effect of the update is small in the existing method.

**Figure 7:**
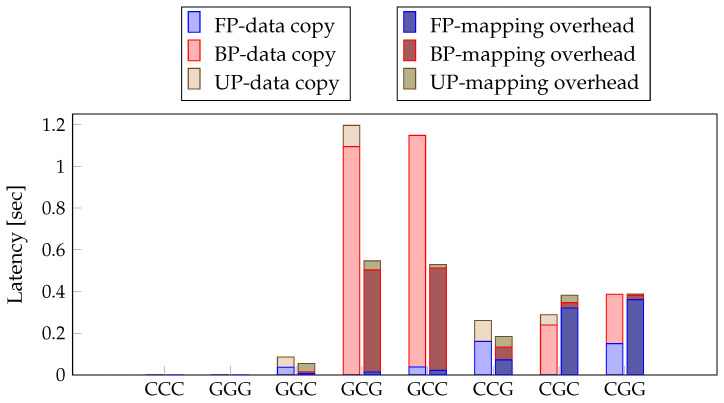
Results of profiling eight cases for data transfer. The three words represent the processor of the FP step, the processor of the BP step, and the processor of the UP step in order. In this study, C means CPU, G means GPU.

For the layer-wise PSM to select the optimal process, we profiled 8 cases of different FP, BP, and UP processors in order, as explained in Section 3.2. We investigated and compared the data transfer times of the method with shared memory and without shared memory to confirm the effectiveness of the method. The method without shared memory copied data between different processors. In contrast, the method with shared memory used mapping/unmapping to access memory. Figure 7 presents the result of profiling the data transfer time of the existing method and the method using unified memory. When the processors of FP and BP are different, such as in the cases of GCG and GCC, more time is required. This is especially true for BP, which was approximately twice as slow when using a different processor. In CGC and CGG, data copying takes more time in BP than in FP, but more mapping overhead is performed in FP than in BP. Additionally, CGC may require less data copying. However, due to the overall performance of the CPU, the overall learning time is longer. Layer-wise PSM selects a combination of processors by layer. The result of profiling latency and data transfer time for each layer is depicted in Figure 6a–c and Figure 7.

**Figure 8:**
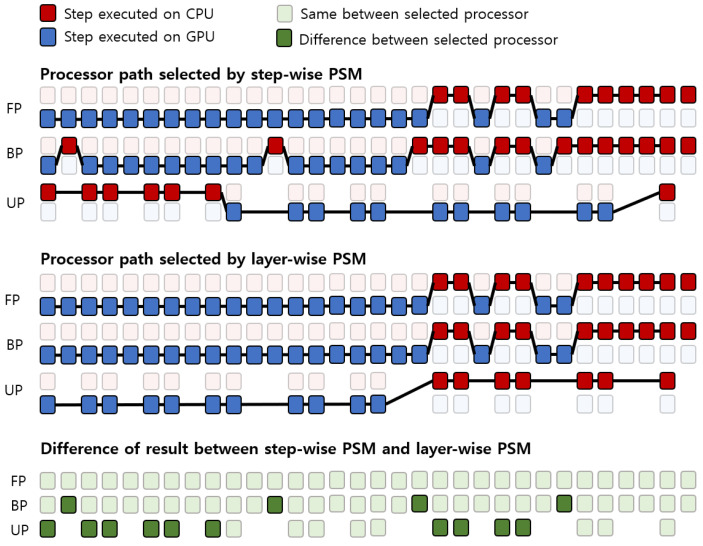
The results of the processor path being selected by step-wise PSM and layer-wise PSM per step, and the difference of selected processor between by step-wise PSM and by layer-wise PSM.

The existing methods [21,22] only compare the latency profile and select a processor at each step. Additionally, the existing methods use their own framework to deploy their method on mobile devices hence, we cannot directly use their method for performance comparison. Therefore, we implemented the existing methods using our framework and these were referred to as the step-wise process selection method. Figure 8 shows the processor path selected by step-wise and layer-wise PSM with a batch of 32. The selected processor by step-wise and layer-wise PSM differs by 4 in the BP steps and by 10 in the UP steps. The processor path selected by the step-wise PSM requires 16 data transfers between steps and 28 data transfers between layers. On the other hand, the processor path selected by the layer-wise PSM only requires 11 data transfers per step. The total amount of data transfer of step-wise PSM is 44, and the total data transfer of layer-wise PSM is 11, which is four times more than that of step-wise PSM. We confirm that the processor path selected by the layer-wise PSM is more consistent and effective than the step-wise PSM. Since the data transfer time is one of the main factors affecting the deep learning training time, we can expect that the proposed method of selecting the processor path by considering the data transfer can yield better performance.

Table 1 shows the latency resulting from the proposed method for deep learning training along with that of single processing and the latency reduction rate. The step-wise and layer-wise PSM without shared memory reduced the latency by 19.68% and 22.78%, whereas the step-wise and layer-wise PSM with shared memory reduced the latency by 22.57% and 27.80%, compared to using the CPU alone. The step-wise and layer-wise PSMs with shared memory have lower latency than those of the two methods not using shared memory. We confirm that the use of unified memory is important in mobile devices. Additionally, the layer-wise PSM is better than the step-wise PSM in both the method with shared memory and without shared memory. Even if it is not a condition of unified memory, a performance improvement can be noted by using the layer-wise PSM on the mobile devices. Analyzing these results, layer-wise PSM with shared memory has the lowest latency.

In the proposed method of profiling latency and data transfer time, the layer-wise PSM is more efficient and consistent than the step-wise PSM. In the maxpool layer, shortcut layer, and weight update, the execution time is smaller than that of the convolutional layer, but by selecting a similar processor in a layer-wise PSM, the locality of the processor is increased. It is effective to consider both the latency and data transfer time of the CPU and GPU. In batch 32, the layer-wise processor algorithm with shared memory reduced the latency by 27.80%, compared to the CPU and by 18.25%, compared to the GPU.

### 4.4. Evalution Experiments

#### 4.4.1. Batch Size

The batch size is an important parameter for deep learning training [52]. As for the inference, one data comes in with a batch of 1, but in training, more memory is required corresponding to the batch size. Thus, in on-device learning, it is important to set the model size and batch size to fit the memory of the device. To see how the batch size affects the deep learning training speed, we investigated the neural network model training time according to the batch size.

**Figure 9:**
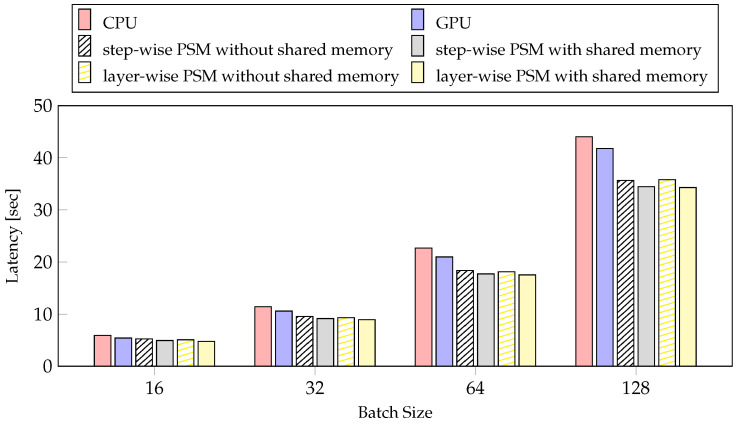
The experiments about latency of PSMs according to the batch size.

Figure 9 presents the experimental results of model training latency with PSMs according to batch size. As the batch is doubled, the amount of calculation and calculation time similarly double. So, the difference in processor execution time of each layer becomes larger. The layer-wise PSM with shared memory has significantly reduced the latency by 28.4% using a batch size of 128 and by 24.42% using a batch size of 16, compared to the CPU. As the batch size increases, the latency reduction ratio increases. The layer-wise PSM with shared memory becomes more effective as the batch size is increased.

**Figure 10:**
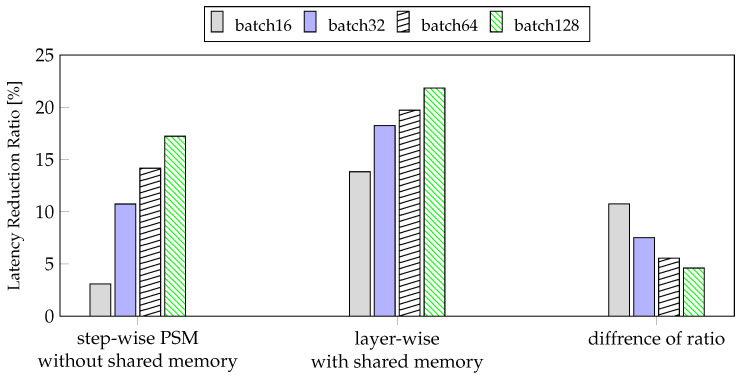
The result of latency reduction ratio of step-wise PSM on method without shared memory and layer-wise PSM with shared memory compared to GPU.

However, in the case of mobile devices, there is a limit to how much the batch size can be increased owing to limited memory. When training a model, we have to adjust the maximum batch size to fit the model size and memory. Therefore, the ratio of latency reduction according to the batch is important when setting the batch size. In Figure 10, we can see that both our method and the conventional method increases the reduction rate as the batch size increases relative to the GPU. However, the latency reduction rate of the proposed method is higher as the batch size is reduced compared to the step-wise PSM without shared memory. Therefore, our method, layer-wise PSM with shared memory, works better with smaller batches than step-wise PSM without shared memory.

#### 4.4.2. Average Power Consumption

**Figure 11:**
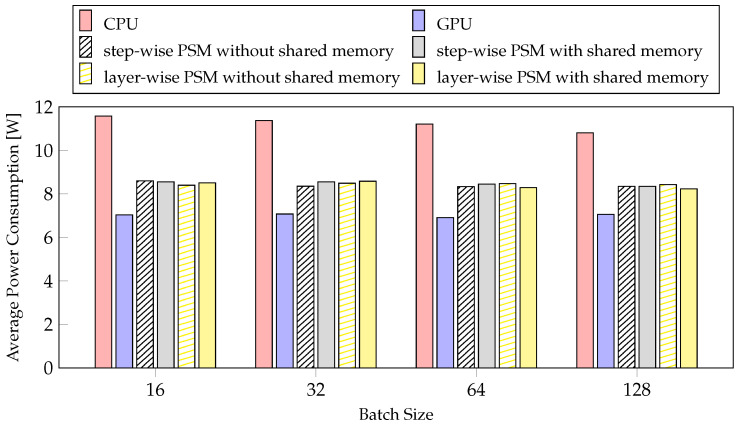
The experiments about average power of PSMs for training per batch size.

Power is an important factor in mobile systems [53]. We measured the average power consumption according to PSM and batch size. In Figure 11, the step-wise PSM without shared memory is the method with the smallest reduction rate, and the layer-wise PSM with shared memory is the method with the largest reduction rate, but the average power consumption is similar. The results of profiling the average power consumption for the two methods while changing the batch size are also similar. The latencies of CPU and GPU differ considerably with the average power consumption. The CPU was selected 27 times, and the GPU was selected 55 times by layer-wise PSM with shared memory, as depicted in Figure 8. These results indicate that the batch size and the proposed method do not affect the average power consumption, but they affect the usage cost of the CPU and GPU. Therefore, the average power consumption is similar, but the larger the batch size, the greater the performance improvement thus, it is better to use a case with a larger batch size. However, on mobile devices, the batch size cannot be increased indefinitely according to the memory and model size. Therefore, considering the average power consumption, our method is better than training the network model using a single processor, regardless of batch size.

#### 4.4.3. Deep Learning Inference

**Figure 12:**
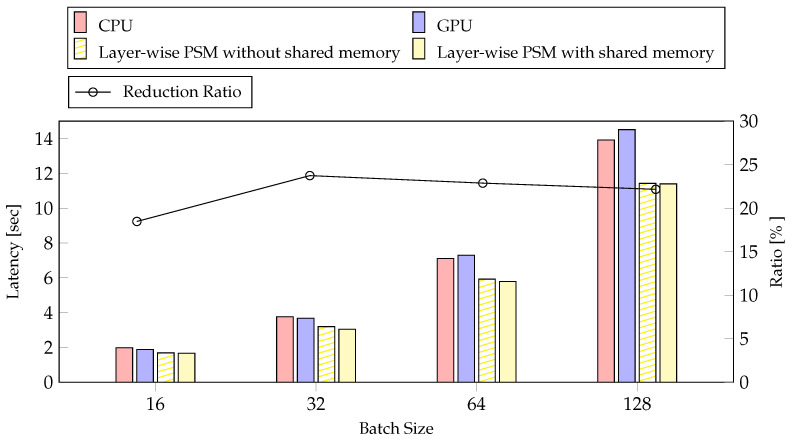
The result of latency executed our method on inference according to batch size. The reduction rate compared our method to the CPU.

We performed experiments with inference to demonstrate the efficiency of our method. We measured latency and average power consumption according to batch size. Figure 12 shows the result of latency and reduction ratio according to batch size. We confirmed that inference takes more time on the GPU than on the CPU when the batch size is large. Inference has almost no data copy overhead because data transfer only needs to transfer the results between steps. Therefore, the difference in performance was not significant between the method with and without the use of shared memory. Nevertheless, the ratio of reduction varied from 2% to 5%. The reduction rate is not proportional to the batch size. In Figure 12, the reduction rate was greatest for batch 32, and decreased with increasing batch size, unlike in training. Our method reduced latency by at most 23.74% with batch 32 and by at least 18.47% with batch 16.

**Figure 13:**
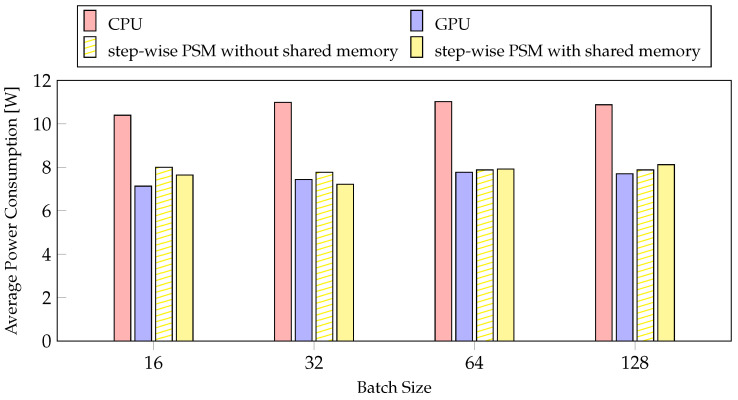
The experiments about average power consumption of PSMs per batch size.

We measured average power consumption to compare layer-wise PSM on inference and training. In Figure 13, the layer-wise PSM with shared memory reduced average power consumption by 36% compared to CPU, and results of layer-wise PSM is similar to that of GPU. This is because the CPU, which consumes a considerable amount of power, is selected for some layers of the FP. The difference in average power consumption between the proposed method and the GPU is smaller in the inference than in the training method. Therefore, the layer-wise PSM is more efficient in inference than in training.

Table 2 shows that although the proposed method improves in inference, it generally shows better performance in training. The maximum reduction in inference is 23.74% with a batch size of 32, and in training, the maximum reduction is 28.40% with a batch size of 128. Even in batch 32, we observed a better performance by 27.80% in training than in inference. The average power consumption compared to the CPU of inference and training decreased by 36.42% and 35.34% on average, respectively. Average power consumption compared to CPU is similar for both inference and training. However, because the inference performance is affected by the processor, the inference is better compared to GPU.

## 5. Conclusions and Discussion

Herein, we proposed layer-wise PSM on unified memory to maximize the usability of the available resources of mobile devices such as the CPU, GPU, and unified memory. By using unified memory on a mobile device, we could prevent memory duplication and eliminate the data copy time during model training. The layer-wise PSM selects a combination of processors suitable for deep learning training using the result of overhead profiling. To verify the effectiveness of on-device learning on mobile devices, we applied the proposed method to ASC, and conducted the experiments in both inference and training. Specifically, we performed an experiment to determine whether the proposed method exhibited a performance improvement if various factors that could affect the on-device learning were changed, including batch size and average power consumption. We performed experiments by varying the batch size and time and average power consumption at the inference to compare whether our method was effective in the inference as well.

Experimental results for training indicated that the layer-wise PSM reduced the latency for model training by 21.84% using a batch size of 128 and 13.83% using a batch size of 16, compared to those measured on the GPU. In addition, the latency reduction rate of layer-wise PSM, compared to the step-wise PSM without shared memory, was 10.75% using a batch size of 16 but 4.6% using a batch size of 128. Based on our results, owing to the limitation of mobile devices to increase the batch size, we can conclude that the proposed layer-wise PSM method with shared memory was more effective than step-wise PSM without shared memory. From the perspective of average power consumption, we confirmed that the batch size and the proposed method did not affect the average power consumption, but they affected the usage of the CPU and GPU. Therefore, on mobile devices, our method was more efficient than step-wise PSM without shared memory, regardless of the batch size. In inference, layer-wise method without shared memory reduced latency by 23.74% compared to CPU. The layer-wise method with shared memory reduced latency by 22.50% compared to GPU. The layer-wise method was effective for both inference and training. The average power consumption for inference was comparable to that of the GPU. In an inference where most of the time-consuming layers were performed on the GPU, the average power consumption compared to the GPU was more efficient than training.

Although the proposed method showed the possibility of accelerating model training for on-device learning, the evaluation was conducted using a limited selection of devices. Therefore, it is necessary to measure the performance by applying it to various devices with different computing resources. In addition, the proposed method has limitations in improving the accuracy of the model by applying simple pruning to the network model. However, we expect that additional performance improvements are possible if the model is made more lightweight by using methods such as knowledge distillation. Currently, ResNet-18 has been applied with pruning, but until now, the model size and the amount of computation have been excessively large for on-device learning. With knowledge distillation, which applies large-sized models to small-sized models, efficient real-time deep learning training on mobile devices can be expected.

## Figures and Tables

**Table 1:** The experiments of training the neural network model with a batch of 32 including latency executed by step-wise PSM and layer-wise PSM and latency reduction rate compared to CPU.

	Latency [s]	Reduction Ratio [%]
CPU	11.43	-
GPU	10.57	8.14
Step-wise PSM without shared memory	9.55	19.68
Step-wise PSM with shared memory	9.25	23.57
Layer-wise PSM without shared memory	9.31	22.78
Layer-wise PSM with shared memory	8.94	27.80

**Table 2:** The reduction ratio of the latency and average power consumption performed by the proposed method compared to by the CPU.

	Latency Reduction Ratio [%]		Average Power Consumption Reduction Ratio [%]	
	Inference	Training	Inference	Training
**Batch 16**	18.47	24.23	36.06	37.83
**Batch 32**	23.74	27.80	36.98	33.89
**Batch 64**	22.88	28.22	37.01	34.32
**Batch 128**	22.17	28.40	35.66	35.33

## Data Availability

Data sharing not applicable.
